# How does sports participation influence individual gender attitudes? An empirical study based on CGSS data

**DOI:** 10.3389/fpsyg.2026.1728590

**Published:** 2026-04-15

**Authors:** Jincao Zhai, Dingwu Liu, Zerong Wang

**Affiliations:** 1 College of Physical Education and Sport Science, Qufu Normal University, Qufu, China; 2 College of Physical Education and Sport Science, Shanxi Datong University, Datong, China

**Keywords:** empowerment, gender attitudes, gender equality, men in sports, sports participation

## Abstract

**Introduction:**

The modernisation of gender attitudes constitutes a fundamental prerequisite for achieving societal gender equality. Recognized as a powerful vehicle for advancing social equity, sports has demonstrated significant potential in fostering gender equality. However, the micro-level interactions between sports participation and individual gender attitudes remain insufficiently explored.

**Methods:**

This study uses data from the 2023 China General Social Survey (CGSS) as the research sample. Multiple linear regression models, robustness tests, instrumental variable (IV) tests, and interaction term tests were conducted to assess the association between sports participation and individual gender attitudes, as well as how this association manifests among the male population.

**Results:**

(1) Sports participation is positively and significantly associated with the modernisation of individual gender attitudes (*β* = 0.853, *p* < 0.01); (2) This positive association is also significant among men, suggesting that sports participation may help them break from the constraints of traditional gender norms (*β* = 0.563, *p* < 0.05); (3) Compared to its association with women’s attitudes, the relationship between sports participation and men’s gender attitudes appears relatively weaker.

**Discussion:**

Our analysis demonstrated that sports participation, as a typical form of embodied practice, has a positive correlation with the modernization of individual gender attitudes. However, this effect exhibits notable gender differences, with the effect on men being relatively weaker than that on women. Future efforts may benefit from optimizing the sporting environment and strengthening institutional reforms within the sports field—such as safeguarding the participation rights of diverse groups, promoting equitable resource allocation, and encouraging the de-gendering of media representation—in order to unlock the greater potential of sports in promoting gender attitudes.

## 1 Introduction

Gender equality constitutes a perpetual theme in humanity’s pursuit of fairness, justice and equity, serving as a barometer of social progress and civilisation, and an essential objective for sustainable human development (The State Council Information Office of the People's Republic of China, 2015). Sport has garnered increasing recognition as a valuable non-policy instrument for advancing gender equality. Since the 1924 Paris Olympics officially recognized women’s right to compete, discussions on gender equality centered around women’s participation in the Olympic movement have flourished. Existing studies generally acknowledge “challenging stereotypes,” “female empowerment,” and “liberation” as mainstream perspectives on how sports promote gender equality (Xiong et al., 2025). In the contemporary context of sport’s continued evolution and institutional expansion, scholarly discourse regarding its role in advancing gender equality has extended well beyond a narrow focus on elite Olympic competition. The field now encompasses a broad spectrum of domains, including school physical education, community-based sport initiatives and sports management (Shaw and Frisby, 2006). Underpinning this expanded inquiry is the conceptual premise that “gender equality in the sports domain influences gender equality beyond it,” a logic that has guided investigations into sport’s distinctive function within contemporary gender relations (Pfister, 2011).

Although these studies have enriched academic understanding of the relationship between sports and gender equality, there remains significant room for further expansion in related research. First, in terms of perspective, existing studies predominantly view sports as a cultural symbol representing fairness and justice, examining how its symbolic significance in challenging gender norms relates to societal gender equality (Tännsjö and Tamburrini, 2000; Huggins and Randell, 2007). However, few studies have directly examined the micro-level link between sports participation and individuals’ gender attitudes. If sports are to function as more than a symbolic force for equality, it is crucial to investigate the intrinsic link between sports participation and individual-level attitudinal change. Establishing this micro-foundational link is a necessary step toward explaining how macro-level social outcomes might emerge. Second, in terms of content, existing studies have predominantly focused on extended topics regarding women’s participation in sports and the evolution of their gender attitudes, while paying insufficient attention to impact of sports participation on men’s gender attitudes (Vaquero-Cristóbal et al., 2024). A pressing question remains: Does sports participation significantly influence men’s gender attitudes? If so, does it promote progressive views or reinforce traditional ones? A deeper exploration of this issue will help overcome the gender perspective limitations in current research and contribute to a more comprehensive theory of gender and sport. Third, most studies to date have been theoretical or focused on paradigm-building, with limited empirical research examining the relationship between sports participation and individual gender attitudes. This constrains the explanatory power of sport as a vehicle for gender equality and hampers the formulation of targeted policies.

In light of these gaps, the present study addresses the following interconnected objectives. First, it empirically examines the relationship between sports participation and individual gender attitudes, utilizing data from the Chinese General Social Survey (CGSS). Second, to interpret this relationship and bridge the micro–macro divide, it proposes and elaborates a conceptual framework centered on “bodily experience–cognitive shift.” This framework offers a systematic lens for understanding how physical engagement might translate into attitudinal change. Finally, with a particular focus on male respondents, the study investigates whether and how this relationship manifests among men, thereby addressing the gender perspective limitation in current research. By combining empirical analysis with theoretical conceptualization, this research aims to provide robust evidence on the sport-gender attitude linkage and advance a more nuanced theoretical understanding of sport’s potential role in fostering gender equality.

## 2 Literature review and research hypotheses

### 2.1 The connotation and measurement of gender attitudes

Gender attitudes, a subsidiary concept to gender equality, are fundamentally distinct from the latter. Gender equality refers to the equal status, rights, resources, and opportunities that all individuals, regardless of gender, should enjoy in political, economic, cultural, social, and family spheres. Achieving gender equality relies on systematic and institutionalized efforts across numerous domains such as law, policy, education, and culture. In contrast, gender attitudes constitute an indispensable internal prerequisite for achieving gender equality and represent the ultimate point of application for the effective implementation of various equality measures. Also referred to as gender role attitudes, they encompass an individual’s overall perceptions regarding role norms, division of labour, and behavioural patterns for men and women (Xu, 2016). They represent not only a personal understanding of gender relations but also an internalized value system that directly influences an individual’s attitudes and behavioural choices in social, familial, and professional contexts. Only when members of society subconsciously develop values grounded in gender equality can gender equality at the conceptual and institutional levels be more deeply translated into reality.

Current academic approaches to measuring gender attitudes can be broadly categorised into three types. The first employs dedicated gender attitude modules within large-scale, comprehensive social surveys. Examples include the World Values Survey (WVS), the International Social Survey Programme (ISSP), the Chinese General Social Survey (CGSS), and the Chinese Women’s Social Status Survey, all of which have been utilised in domestic research (Yuan et al., 2022). The second type relies on composite “gender equality indices” from international organisational reports to gauge the development level of gender attitudes. A prominent example is the Gender Inequality Index from the UN Human Development Report, which assesses a region’s level of gender equality across three dimensions: reproductive health, empowerment, and economic status (Bhardwaj, 2022). The third type is a qualitative approach involving the construction of typological models based on in-depth interviews and theoretical insight. Building on a profound analysis of the underlying logic behind the formation and evolution of gender attitudes, combined with field interview data, Hochschild classified gender attitudes into three distinct types: traditional, transitional, and egalitarian (Dai, 2023). This study adopts the widely cited gender attitude module from the CGSS questionnaire to measure individual gender attitudes and analyse their relationship with sports participation.

### 2.2 Factors influencing gender attitudes

The factors shaping an individual’s gender attitudes toward traditional or modernorientations are multifaceted and complex. Recent research has explored their driving mechanisms from various perspectives. Such as, (1) demographic analysis of direct and mediating variables affecting subjective gender attitudes. A body of research indicates that socio-demographic variables such as gender, ethnicity, religious affiliation, educational attainment, and number of children are significantly associated with individual gender attitudes (Fan, 2003). (2) Investigating the impact of digital technology, exemplified by the internet, on gender attitudes. Technological change is considered closely linked to modernisation in individual gender attitudes. Young women’s use of digital platforms to express views and assert rights transcends the boundaries of traditional online spaces, challenges male-oriented technological narratives, and fosters a more profound societal understanding of gender issues (Wang and Zhu, 2024). (3) Research revealing the constructed nature and process of gender attitude formation. Theoretical frameworks such as feminist epistemology, life course theory, the separation of public and private spheres, and social constructivism have been pivotal in explaining the formation of attitudes. These studies generally posit that perceptions of men’s and women’s roles in political and economic life are forms of constructed knowledge. This construction is not static but evolves dynamically alongside an individual’s lived experiences (Pérez-Díaz et al., 2025). Policy frameworks, socio-cultural norms, early life experiences, significant life events, and the educational backgrounds of individuals and their parents collectively form the foundation upon which gender attitudes are constructed. (4) Interpreting the historical trajectory of gender attitudes from traditional to modern. Relevant studies posit that societal development is inherently linked to individual values (Blee and Tickamyer, 1995). However, with accelerated industrialisation and urbanisation, recent research has questioned the linear progression of gender attitudes, noting a trend of reverting to traditionalism in certain dimensions. For instance, Yang Juhua et al. found that while overall societal gender attitudes trend toward modernity and equality, specific aspects, such as roles within the occupational division of labour, show varying degrees of regression from modern to traditional norms (Yang et al., 2014).

Sport as a significant cultural field, provides a dynamic arena for the reshaping of individual gender attitudes. Given the constructed nature of gender attitudes and the contemporary context of a potential regression toward traditional norms, investigating the influence of sports participation on the individual gender attitudes holds substantial practical significance.

### 2.3 A proposed framework: bodily experience, cognitive shift, and egalitarian habitus

As a crucial mechanism for individual socialization, sports participation is recognized as a significant field for deconstructing “old gender role norms” and reconstructing “new gender value systems.” Substantial research confirms that sports participation is recognized as an important context for the development of gender attitudes (Gardella, 2023). The United Nations Educational Scientific and Cultural Organization (UNESCO) also emphasises that sports participation plays a decisive role in raising awareness and challenging entrenched gender norms, significantly shaping perceptions and attitudes toward gender roles (Yan and Wen, 2025). Based on existing literature, this study attempts to construct a “Bodily Experience–Cognitive Transformation–Social Wellbeing” theoretical framework, aiming to provide a clear and systematic analytical perspective and theoretical reference for understanding the relationship between sports participation and gender attitudes.

The fundamental reason why sports participation influences individual gender attitudes lies in its direct engagement with the body—the very site where gender norms are inscribed. Traditional gender attitudes discipline the body—through constraints on posture, expressions of strength, and physical space—thereby solidifying men and women into specific gendered divisions of labour (Cole, 2000). This physical differentiation further reinforces stereotypical gender norms (Yuan et al., 2022). Sports practice offers a unique, degendered physical language. When a woman throws a punch in the boxing ring or a man leaps in figure skating, they are not merely engaging in a sport; they are challenging, through their bodies, the binary narrative that “men should be strong and aggressive, women gentle and submissive.” This bodily contestation is regarded as a potential starting point for attitudinal change. Gender schema theory suggests that the sports field provides a practical arena in which traditional gender schemas can be rewritten (Barragan, 2015). Here, the body is no longer a passive “object” subjected to social discipline, but becomes an active “subject” challenging gender boundaries. Research on women’s participation in strength training has also observed that through cross-gender displays of strength and flexibility, individuals challenge the presuppositions of traditional gender segregation at the level of bodily schema. This process offers a micro-level bodily dimension for understanding how sports shape gender attitudes (Xu and Hu, 2025).

The breakthrough in bodily practice does not occur in isolation; it may trigger a chain reaction at the cognitive level. When individuals engage in sports that are gendered as “atypical,” their bodies undergo experiences that conflict with prevailing social expectations. Such embodied practice is theorized as a critical juncture for individuals to re-examine gender boundaries. In particular, the sensations of strength, coordination, or grace perceived through the body constitute “first-hand evidence” that refutes social discipline. This bodily evidence reconstructs and reshapes personal identity, enabling individuals to construct and negotiate self-identity, social understanding, and modes of interaction with the environment through reflection on bodily experience (Schultz, 2018; Xiong, 2025). Drawing on Kane’s “sports continuum” theory, Janet S.’s research on mixed-gender basketball demonstrates that competitive encounters in co-ed settings allow men to physically perceive women’s competitiveness, thereby providing an important cognitive pathway for revising their entrenched beliefs about gendered abilities (Fink et al., 2018). At this juncture, sport functions as a cognitive catalyst: it transforms the abstract ideal of gender equality into a tangible, perceptible bodily reality, thereby enabling individuals to move from the myth that “gender determines ability” toward a cognitive restructuring where “ability transcends gender.” This potential extends beyond the re-evaluation of women’s capabilities, positively influencing the reconstruction of masculinities, the inclusion of sexual minorities, and the acceptance of transgender individuals (Anderson, 2010).

The sustained interaction between bodily contestation and cognitive reflection ultimately externalizes into a stable disposition toward egalitarian consciousness—a process that epitomizes what Bourdieu conceptualizes as the formation of habitus (Li, 2021). What distinguishes sports participation is its capacity, through the medium of physical practice, to impel individuals to reflect upon pre-existing gender norms, thereby furnishing a critical space for the emergence of a new habitus. Furthermore, repetitive and sustained physical training institutionalizes the cycle of “bodily practice–cognitive reflection” (Gorely et al., 2016). Through continuous reinforcement, this cycle is transmuted into bodily and cognitive capital that resists traditional gender norms. This capital, accumulated through persistent and repeated engagement, becomes internalized as a habitus oriented toward fairness and inclusivity. Ultimately, it transcends the boundaries of the sports field, crystallizing into a practical logic that guides individuals to uphold principles of equality and respect across multiple domains—including professional, familial, and everyday life (Brown, 2005; Thorpe, 2009; Jejeebhoy et al., 2017). As the British scholar David Brown elucidates, physical practice somatizes the egalitarian habitus; through repeated performance, individuals gradually forget the historical process by which this habitus was inculcated, thereby naturalizing it. It eventually becomes anchored within social structures, serving as a critical instrument for challenging gender orthodoxy. In this sense, the sports field can be understood as a potential incubator of habitus. Through embodied learning on the playing field, individuals may carry the seeds of equality back into the broader social structure—a dynamic that suggests sports participation may facilitate a transformative leap from bodily liberation to social action (Zhang, 2021).

In summary, the shaping of individual gender attitudes through sports participation constitutes a complex process wherein bodily practice triggers cognitive reconstruction, ultimately culminating in the internalization of an egalitarian habitus. Within the sporting arena, individuals acquire embodied experiences that challenge gender binaries by confronting gendered bodily norms. The cognition catalyzed by such experiences impels individuals to reassess presuppositions regarding gender-based capabilities, while prolonged and repetitive training solidifies these reflections into a durable disposition that guides everyday action.

Based on this, Hypothesis 1 is proposed: sports participation is positively associated with the modernization of individuals’ gender attitudes.​.

### 2.4 The impact of sports participation on men’s gender attitudes

It is worth noting that exploring the impact of sports participation on gender perceptions cannot be separated from a constructive dialectical logic. “Constructive” here means that the direction of construction is not a one-way linear process, but presents a profound duality sports participation can be both a progressive space for challenging gender perceptions and promoting ideological change, and a conservative force that reinforces or reproduces inherent power inequalities.

In recent years, with the growing global emphasis on and vigorous advocacy for sports participation in advancing gender equality, scholars have gradually turned their attention to the tension between the evolution of gender relations in the sports field and individuals’ gender perceptions. A consensus has been reached among many scholars that the role of sports participation in advancing the modernisation of women’s gender perceptions is relatively direct and effective. Whether it is breaking physical discipline to demonstrate female strength, or competing for discourse power through professional events, these facts have proven the great potential of sports participation in empowering women to challenge the “gaze” of gender orthodoxy. However, the situation for men is more complex, and some studies have even pointed out that there is a certain contradiction (Ogilvie and McCormack, 2021).

On the one hand, sports participation is associated with men breaking free from the constraints of traditional gendered masculinity, cultivating gender awareness such as emotional expression, teamwork and respect for differences, and is linked to the modernisation of their gender perceptions. M. F. Ogilvie explored the relationship between team sports and university students’ gender perceptions, noting that when men train and compete alongside women, gender opposition is weakened, and men’s understanding of strength, competition and cooperation is reconstructed. This shift is associated with a move from a dominant role to one characterized by greater empathy and social responsibility, thereby contributing to dynamic balance in gender relations through micro-level practice (Moura, 2021). Eva Soares Moura incorporated men’s experiences into the analytical framework of sports for gender equality, and found that sports participation is associated with enhanced cognitive sensitivity to gender equality issues and sense of self-awareness among men. The study also highlighted that valuing and leveraging men’s role as “equality advocates” in the sports field plays a significant role in building a more inclusive and equal gender culture in society (Guerrero and Guerrero Puerta, 2023).

Miguel A. et al., meanwhile, examined the relationship between sports intervention measures and gender equality awareness among adolescents aged 6 to 14. Their findings indicated that sports activities provide a more inclusive and fair environment for development, which is associated with increased enjoyment of sports among female adolescents, as well as lower levels of gender-discriminatory attitudes and behaviours among male adolescents (Oxford, 2019).

Based on this, Hypothesis 2: sports participation has a significant promoting effect on the modernisation of men’s gender perceptions.

On the other hand, sports typically take place in highly competitive and confrontational environments, where activities that emphasise strength, endurance and physical contact are often ascribed higher “masculine” value. This structural bias further reinforces traditional gender norms centered on male dominance, physical prowess and emotional restraint, solidifying men’s stereotypical perceptions of their own gender roles (Yang, 2024). Furthermore, the significant gender inequality that exists within the sports field continuously strengthens the ideology of male hegemony at both institutional and cultural levels. Its specific manifestations include gender segregation in organisational structures, gender-based disparities in resource allocation, gender bias in media representation, and homophobic discourse—all of which, to a certain extent, reproduce and rationalise “masculinity” (Berg et al., 2023). Some scholars have further pointed out that the current discourse advocating for equality and rights in women’s sports participation, despite appearing progressive on the surface, essentially frames men as inherently individualistic paragons of competitiveness and agency in its underlying logic. Fundamentally, this constitutes a form of flexible and moderate hegemony (Işıkgöz et al., 2025).

Based on this, Hypothesis 3: sports participation has a significant inhibiting effect on the modernisation of men’s gender perceptions.

## 3 Methods

### 3.1 Data source

This study utilizes data from the 2023 wave of the Chinese General Social Survey (CGSS), which provides comprehensive coverage of demographic characteristics, sports participation, and gender role attitudes. The initial sample consisted of 11,326 respondents. Missing values across all variables were addressed through multiple imputation using chained equations. Diagnostic analysis of missing patterns confirmed that data were missing at random relative to observed covariates, supporting the application of this method. Twenty imputed datasets were generated based on a complete information matrix incorporating all study variables and auxiliary predictors. All analyses incorporated official CGSS sampling weights and accounted for the complex survey design by clustering standard errors at the primary sampling unit level. After pooling imputed datasets, the final analytical sample comprised 5,770 complete cases.

### 3.2 Variable selection

#### 3.2.1 Dependent variable

Gender attitudes were measured using the CGSS questionnaire item set A42, which consists of the following five Likert-scale statements: (1) “Men should focus on careers, while women should focus on families”; (2) “Men are naturally more capable than women”; (3) “For a woman, marrying well is more important than having a successful career”; (4) “During economic downturns, female employees should be laid off first”; and (5) “Husbands and wives should share household chores equally.” With the exception of the fifth statement, all items reflect traditional gender role attitudes. The data were processed as follows. First, all items were reverse-coded (1 = “strongly agree,” 5 = “strongly disagree”) so that higher scores indicate more egalitarian gender attitudes. Item and scale analyses were then conducted. The item-rest correlation for the fifth item (“Husbands and wives should share household chores equally”) was −0.027, substantially lower than those of the other items (range: 0.530–0.620). Moreover, its content direction was inconsistent with the dimension of traditional attitudes measured by the remaining four items. Consequently, this item was excluded from further analysis. Reliability analysis of the remaining four items yielded a Cronbach’s *α* of 0.732, indicating acceptable internal consistency. The Kaiser–Meyer–Olkin measure was 0.745, and Bartlett’s test of sphericity was significant (*p* < 0.001), confirming the suitability of the data for factor analysis. Principal component analysis was performed, and one common factor was extracted based on the eigenvalue-greater-than-1 criterion, accounting for 55.51% of the total variance. Factor loadings ranged from 0.650 to 0.806, demonstrating good structural validity. To enhance interpretability and avoid treating ordinal variables as continuous, the extracted factor scores were standardized into a 0–100 index using the following equation: Standardized Index = (Original Factor Score − Minimum Score)/(Maximum Score − Minimum Score) × 100. The resulting continuous variable was labeled “Gender Egalitarianism Attitude,” with higher scores reflecting more progressive gender attitudes.

#### 3.2.2 Independent variable

The independent variable was sports participation. This was measured using a Likert scale item from the CGSS questionnaire that inquired about the frequency of the respondents’ sports activities over the past 12 months. The original response options were: “Daily = 1,” “Several times a week = 2,” “Several times a month = 3,” “Once or a few times a year = 4,” and “Never = 5.” To ensure directional consistency in measurement (where a higher value indicates a more positive or frequent behavior), the responses were reverse-coded as follows: “Daily = 5,” “Several times a week = 4,” “Several times a month = 3,” “Once or a few times a year = 2,” and “Never = 1.” In line with standard practice in quantitative social science research (Frankfort-Nachmias et al., 2020), this ordinal variable was treated as continuous in the primary analyses. This widely accepted and effective compromise facilitates the use of more interpretable parametric statistical models, such as linear regression.

#### 3.2.3 Control variables

To mitigate omitted variable bias in causal inference, this study systematically incorporates theoretically grounded, multidimensional control variables into the model. These variables span the following domains: (1) Structural demographic characteristics—including sex, ethnicity, age, and marital status—to account for systematic differences arising from social identity and life-cycle stage; (2) Socioeconomic status—measured by educational attainment and household economic condition—to control for confounding effects related to resource accessibility and social-class position; (3) Key behavioural and contextual exposures—proxied by frequency of internet use—to capture the potential influence of digital access on attitudes and behavioural patterns. The inclusion of these variables is designed to block major confounding pathways from fundamental social structures to the outcomes of interest, thereby strengthening the internal validity of the estimates for the core independent variable. Specific coding details are presented in Table 1.

**Table 1:** Variable definitions.

Variable type	Variable name	Variable definition
Demographic characteristics	Gender	Male = 1
		Female = 0
	Ethnicity	Ethnic minority = 1
		Han = 0
	Religious belief	No religious belief = 1
		Has religious belief = 0
	Educational level	Below college = 0
		College and above = 1
	Marital status	Married (first marriage with spouse, separated but not divorced, remarried with spouse) = 1
		Single (divorced, widowed, cohabiting, never married) = 0
	Household economic	Far below and below average = 1
		Average = 2
		Above and far above average = 3
	Internet use	Never, rarely, sometimes = 0
		Often, very frequently = 1

### 3.3 Model specification

A multiple linear regression model was employed to examine the impact of sports participation on individual gender attitudes. The model is specified as follows:


$${\text{Gender}}_{i}=\beta_{0}+\beta_{i}{\text{sport}}_{i}+\sum_{i=2}^{n}\beta_{i}\chi_{i}+\varepsilon_{i}$$

In the model, Gender Attitudes*_i_* denotes the gender attitudes of individual *i*, sports participation*_i_* represents their level of sports participation, and $\chi_{i}$ signifies the vector of control variables. $\beta_{i}$ and $\varepsilon_{i}$ denote the constant term and the error term, respectively. To further analyse the specific effect of sports participation on men’s gender attitudes, an interaction term between the gender dummy variable and sports participation was incorporated into the baseline model. This provides an initial test of whether the effect is statistically significant for men. The extended model is specified as follows:


$${\text{Gender}}_{i}=\beta_{0}+\beta_{i}{\text{sport}}_{i}+\beta_{2}{\text{sport}}_{i}\times {\text{gender}}_{i}\sum_{i=3}^{n}\beta_{i}\chi_{i}+\varepsilon_{i}$$

Subsequently, a subgroup regression was conducted exclusively on the male sample to precisely estimate the direction and magnitude of the relationship. The model for this subgroup analysis is:


$${\text{Gender}}_{i\mathrm{men}}=\beta_{0}+\beta_{i}{\text{sport}}_{i\mathrm{men}}+\sum_{i=2}^{n}\beta_{i}\chi_{i}+\varepsilon_{i}$$

### 3.4 Key elements of the identification strategy (IV)

The baseline regression results may be subject to endogeneity bias due to reverse causality. On the one hand, participation in sports may challenge and reshape individuals’ gender attitudes; on the other hand, individuals with more egalitarian gender attitudes may be more inclined to actively engage in sports, which symbolizes the breaking of traditional gender boundaries. To identify the causal effect of sports participation, this study employs an instrumental variable two-stage least squares approach. We select “frequency of attending live sports events in the past year” as the instrumental variable for “individual sports participation.” The validity of this instrument rests on the following theoretical grounds: (1) Relevance: Interest in sports serves as a common intrinsic motivation that drives individuals both to watch and to participate in sports. The experience of watching live events can enhance understanding and enthusiasm for sports, thereby directly encouraging participation; conversely, sports participants are also more likely to be spectators. A close theoretical and empirical connection thus exists between the two. (2) Exclusion restriction (exogeneity): First, from the perspective of behavioural genesis and experiential difference, “attending live events” is a situational cultural consumption activity characterized by its occasional, brief, and passive nature. This contrasts fundamentally with the embodied, repetitive, and proactive practice required for “sports participation.” The former is unlikely to trigger the core mechanism through which sports participation may reshape gender identity via bodily practice. Second, in terms of the temporal scale and intensity of socialization influence, the formation and evolution of individual gender attitudes are primarily embedded within long-term, institutionalized socialization contexts. The transient cultural setting encountered during live spectating is only weakly linked to the deep, long-term socialization processes that shape individuals’ core gender-role attitudes. Therefore, it is reasonable to assume that attending live sports events influences the dependent variable indirectly through its effect on the level of sports participation, thereby satisfying the exogeneity requirement.

## 4 Results

### 4.1 Descriptive statistics

Descriptive results (Table 2) indicated that in terms of gender distribution, approximately 45% of the sample were male and 55% were female. Regarding marital status, the majority of respondents were married (72%), encompassing individuals in their first marriage, those who were separated, or remarried. In terms of educational attainment, 23% held an associate degree or below, while 77% held an associate degree or above, suggesting that the majority of residents in the valid analytical sample had a relatively high level of education. Furthermore, the mean score for gender attitudes was 57.52 (SD = 24.18), indicating a sample-level tendency toward moderately modern and egalitarian gender attitudes. The mean score for sports participation was 2.6 (SD = 1.63), suggesting that the overall level of regular physical activity engagement within the sample was not high.

**Table 2:** Descriptive statistics.

Variable name	Mean	SD	Median	Min	Max
Gender attitudes	57.52	24.18	57.18	0	100
Sports participation	2.6	1.63	2	1	5
Gender	0.45	0.48	0	1	5
Ethnicity	0.09	0.29	0	0	1
Religious belief	0.91	0.28	1	0	1
Educational level	0.23	0.42	0	0	1
Marital status	0.72	0.45	1	0	1
Household economic	1.63	0.59	2	1	3
Internet use	0.64	0.48	1	0	1

### 4.2 Test of regression model

Six regression models were used to analyse the relationship between sports participation and individuals’ gender attitudes. The results are shown in Table 3.

**Table 3:** Regression coefficient and significance of the model.

Variable	Model 1	Model 2	Model 3	Model 4	Model 5	Model 6
	Gender attitudes	Gender attitudes	Dimension 1	Dimension 2	Dimension 3	Dimension 4
Sports participation		0.853*** (0.57)	0.036** (0.041)	0.033** (0.039)	0.022** (0.026)	0.059*** (0.086)
Gender	−3.99** (−0.082)	−4.192*** (−0.086)	−0.341*** (−0.12)	−0.159*** (−0.057)	−0.005 (−0.002)	−0.151*** (−0.067)
Ethnicity	−1.316 (−0.016)	−1.079 (−0.13)	−0.128** (−0.26)	−0.141** (−0.03)	0.105 (0.022)	−0.035 (−0.009)
Religious belief	1.192 (0.014)	1.462 (0.17)	0.114 (0.022)	0.062 (0.012)	0.036 (0.007)	0.031 (0.008)
Educational level	13.99*** (0.244)	13.599*** (0.237)	0.732*** (0.216)	0.604*** (0.184)	0.545*** (0.166)	0.364*** (0.136)
Marital status	−3.583*** (−0.066)	−3.472*** (−0.064)	−0.252*** (−0.080)	−0.122** (−0.04)	−0.16*** (−0.052)	−0.014 (−0.005)
Household economic	1.373** (0.034)	1.186** (0.29)	0.047 (0.02)	0.063** (0.027)	0.104*** (0.045)	0.006 (0.003)
Internet use	9.515*** (0.189)	9.07*** (0.181)	0.556*** (0.188)	0.401*** (0.14)	0.289*** (0.101)	0.3*** (0.128)
Constant term	49.4***	47.6***	2.393***	2.732***	2.527***	3.692***
*N*	5,770	5,770	5,770	5,770	5,770	5,770
*R* ^2^	0.139	0.141	0.139	0.082	0.057	0.062

***p < 0.01, **p < 0.05. The same notation applies throughout.

Model 1 indicates that gender, educational attainment, marital status, family economic status, and internet use are significant factors influencing individual gender attitudes, a finding consistent with existing research (Lanhua and Yao, 2021).

The results of Model 2 show that the coefficient for sports participation is 0.853 and is statistically significant at the 1% level. This suggests that, after controlling for other influencing factors, sports participation is positively associated with an individual’s gender attitudes score.

Results from Models 3 to 6 demonstrate that sports participation is significantly positively associated with all four constituent dimensions of gender attitudes. The strongest effect is observed on dimension 4, while the association with dimension 3 is relatively the weakest. Thus, Hypothesis 1 is supported.

### 4.3 IV tests

To address potential endogeneity concerns between sports participation and gender attitudes, this study employs a two-stage least squares (2SLS) estimation approach. The instrumental variable (IV) estimation results are reported in Table 4.

**Table 4:** IV tests.

Variable	Two-stage least squares (1)		Re-specified independent variable (2)
	Sports participation	Gender attitudes	Gender attitudes
Sports participation		3.276*** (0.895)	3.207*** (0.066)
Attending live sports events	0.504*** (0.208)		
Control variables	Controlled		Controlled
Constant term	42.552		
*F*-statistic	114.734		
*N*	5,770		5,770
*R* ^2^	0.138		0.135

***p < 0.01.

The first-stage regression results show that the F-statistic for the instrumental variable “attending live sports events” is 114.734, well above the conventional threshold of 10 for weak instrument tests, indicating that the selected instrument satisfies the relevance condition and that weak instrument bias is unlikely.

In the second stage, after accounting for potential endogeneity, the estimated coefficient of sports participation on gender attitudes is 3.276 and remains statistically significant at the 1% level. This result aligns with the baseline regression in direction and shows a larger effect size, suggesting that the positive association of sports participation on individuals’ gender attitudes remains robust after controlling for endogeneity. Thus, the main findings are further validated.

### 4.4 Robust tests

The study tested the reliability of the research findings by altering the operationalization of core variables and using data from different years. The specific procedures are as follows:

(1) The coding for the independent variable was changed to “Never, Once or a few times a year” = 0, indicating non-participation in sports; “Daily, Several times a week, Several times a month” = 1, indicating participation in sports.
(2) For the core dependent variable, the method of summing and taking the average was used to replace the previous standardization approach.
(3) The study used data from 2018, 2021, and 2023 for separate analyses. The rationale for selecting these 3 years is that they represent distinct societal and living conditions: 2018 was the year before the onset of the pandemic, 2021 coincided with a more severe phase of the pandemic, and 2023 marks the first year following the pandemic. By comparing the effect of sports participation on individual gender attitudes across these three distinct periods, it is possible to control for potential year-specific anomalies and test the robustness of the relationship between sports participation and gender attitudes over time.

The results are presented in Table 5. The findings show that the effect of sports participation on individual gender attitudes did not change significantly, indicating that the baseline regression results are reliable.

**Table 5:** Robustness tests.

Variable	Re-specified dependent variable (1)	Alternative dataset 2018 (2)	Alternative dataset 2021 (3)
	Gender attitudes	Gender attitudes	Gender attitudes
Sports participation	0.034*** (0.056)	0.764*** (0.058)	1.038*** (0.071)
Control variables	Controlled	Controlled	Controlled
*N*	5,770	5,770	5,770
*R* ^2^	0.147	0.119	0.19

***p < 0.01.

### 4.5 The impact of sports participation on men’s gender attitudes

Employing a stepwise analytical strategy, we examine whether the effect of sports participation on gender attitudes differs by gender. First, we introduce an interaction term between sports participation and gender to test for a statistically significant differential effect. Second, we conduct subgroup analysis on male respondents to clarify the direction and magnitude of the relationship specifically among men.

Results are presented in Table 6. Model 1 indicates that, after controlling for demographic characteristics, the effect of sports participation on gender attitudes differs significantly between men and women (interaction coefficient = 1.739, *p* < 0.01). The male-subsample regression in Model 2 further shows that sports participation is significantly positively associated with the modernisation of men’s gender attitudes, with each additional unit of sports participation associated with a 0.563-unit increase in the gender-attitudes score (*p* < 0.01), supporting Hypothesis 2.

**Table 6:** The impact of sports participation on men’s gender attitudes.

Variable	Model 1	Model 2
	Gender attitudes	Gender attitudes (male)
Male (female = 0)	−1.234 (−0.025)	
Sports participation	1.379*** (0.093)	0.563** (0.04)
Interaction term: sports participation × gender	−1.129** (−0.081)	
Control variables	Controlled	Controlled
Constant term	46.282***	46.742***
*N*	5,770	2,597
*R* ^2^	0.142	0.089

**p < 0.05, ***p < 0.01.

**Figure 1:**
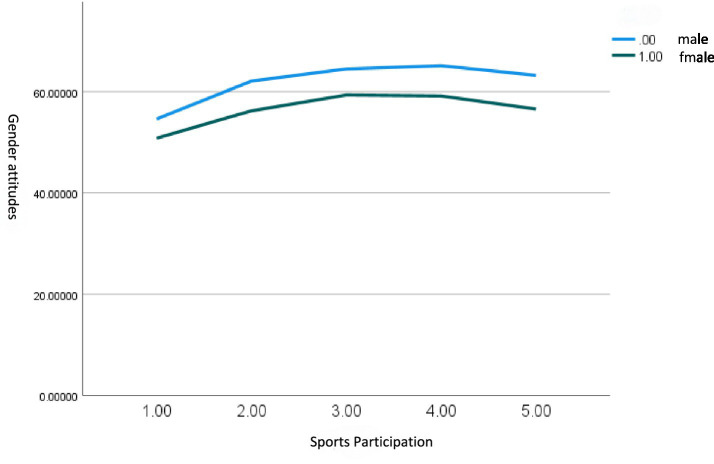
Gender differences in the impact of sports participation on gender attitudes.

The positive interaction term, together with the visual evidence from the fitted curves in Figure 1, indicates that the effect of sports participation is stronger for women than for men. While both slopes are positive, the steeper slope for women suggests a pattern of “gender asymmetry”: sports participation is an effective pathway for modernising gender attitudes for both genders, but its utility in promoting attitude change among men appears comparatively weaker and may warrant further enhancement.

## 5 Discussion

Gender attitudes are a core indicator for measuring a society’s equality and progress in its ideas, culture, and structure. Investigating how sports participation interacts with individual gender attitudes at a micro level helps clarify the systematic value of sports in promoting broader gender equality. Based on a review of existing literature and using CGSS2023data for empirical testing, this paper arrives at the following conclusions:

Firstly, the empirical analysis in this study confirms that sports participation is significantly and positively associated with the modernization of individual gender attitudes. Specifically, compared to individuals who do not participate in sports, those who engage in sports exhibit more progressive gender attitudes, demonstrating higher levels ofrecognition and support for gender equality values. This finding is highly consistent with conclusions from existing studies (Meyer and Roche, 2017). Whether as a daily leisure activity or a practical carrier of resocialization in physical education, sports are widely recognized as having significant potential to enhance awareness, challenge entrenched gender norms, and shape gender attitudes characterized by respect, inclusivity, fairness, and justice [United Nations, 2003; Sport for Development and Peace International Working Group Secretariat (SDPIWGS), 2007]. Moreover, this role manifests uniquely across different countries and regions, demonstrating a wide-ranging influence (Lyras and Hums, 2009). The spiritual core of sports—encompassing solidarity, inclusivity, fairness, and justice—along with their institutional foundations, provides a crucial basis for interpreting the value of sports in promoting gender equality (Pfister, 2011). Existing research has revealed the theoretical implications of sports participation for individual gender attitudes from multiple dimensions, such as the philosophical meaning of the sporting body, embodied reflection in sports, the paradigm construction of the sports field, and the diverse social values of sports (Mahony et al., 2010; Meng, 2011; Yin et al., 2021). Building on this theoretical consensus and integrating the core characteristics of sports practice, sports participation—as a quintessential form of embodied practice—exerts its influence on gender attitudes primarily at the bodily level. Unconventional bodily experiences at this level generate tension with traditional gender norms, which catalyzes the formation of critical cognition. This cognitive reflection is subsequently strengthened and consolidated through sustained training, gradually evolving into an internalized egalitarian habitus. This habitus, in turn, becomes a form of capital for challenging traditional gender role norms and is ultimately manifested and enacted in broader social practice. As Brown’s research indicates, higher frequency of sports participation increases individuals’ exposure to unconventional bodily experiences, thereby positioning regular sports engagement as a potential catalyst for challenging traditional gender stereotypes (Brown et al., 2016). This logic aligns closely with and mutually reinforces the theoretical framework that this study seeks to construct—namely, the “Bodily Experience–Cognitive Reflection–Social Wellbeing” framework. This theoretical framework systematically delineates the interactive logic between sports participation and individual gender attitudes, thereby actively responding to the theoretical imperative of harnessing sports for gender equality and value-led development.

Secondly, sports participation is closely associated with the modernisation of men’s gender attitudes. Grounded in R. W. Connell’s theory of “multiple masculinities,” men are also victims of traditional gender norms, often facing stigmatisation for deviating from societal role expectations. Within this context, men’s sports participation traditionally coded as ‘feminine’ (e.g., dance, gymnastics) holds significant emancipatory potential for identity. Such sports participationhelps may contribute to break through the stereotypes of competition and emotional suppression emphasised by hegemonic masculinity, while also fostering the diversification and humanisation of male identity, thereby carving out a broader space for emotional and behavioural expression for men (Xiong, 2025). On the other hand, women’s sporting experiences exert a reshaping influence on men’s gender attitudes. The exceptional strength and skill demonstrated by women in traditionally male-dominated sports powerfully challenge the biological essentialist biases long held by men. This direct “competence shock” compels male participants to re-evaluate the presumed inherent links between gender, physical prowess, and skill (Scheadler and Wagstaff, 2018). The research by Vezzali et al., resonates with the conclusions of this study from the perspectives of media communication and social cognition. Vezzali et al. noted in their work that the demonstration of women’s athletic abilities is significantly correlated with men’s breakthrough from rigid gender attitudes, and highlighted that this process is amplified via the influence of the mass media (Vezzali et al., 2023). This suggests the potential value of “cognitive reflection,” triggered by observing the exceptional performance of female athletes, in shaping men’s gender attitudes. Furthermore, systematic institutional reforms related to sports participation are undermine male-dominated gender hierarchies from within (Meier, 2000). Such as institutional safeguards for the sporting rights of women and children, respect for the human rights of transgender athletes, and active support for women in leadership roles. These measures collectively signal values of equality, inclusion, and diversity to all members of society, particularly men, encouraging the gradual cognitive and behavioural acceptance and internalization of more inclusive gender attitudes (Anderson, 2010). In summary, sports participation fosters more egalitarian gender attitudes among men through a combination of intrinsic self-liberation, extrinsic role model influence, and institutional normative guidance.

It is worth further reflection that, while this study identified a net positive effect of sports participation on modernising men’s gender attitudes, theoretical literature also suggests potential pathways through which the sports field may reproduce traditional masculinity and reinforce gender biases. The present findings did not support such a constraining pathway. This may be due to several contextual and methodological factors. First, the sample in this study primarily involved mass participation and community-based sports, environments that tend to emphasise health, social interaction, and personal development rather than the reinforcement of hegemonic masculinity, which is often more pronounced in highly commercialised, competitive elite sports settings. Second, under the current socio-cultural backdrop, institutional reforms promoting gender equality in sports—such as safeguards for the participation rights of women, children, and sexual minorities, as discussed earlier—are exerting a broad normative influence that may partly counteract the conservative tendencies inherent in some sporting contexts. Finally, the “cognitive shock” experienced by male participants through exposure to gender-atypical sports or high-performing female athletes could itself act as a potent mechanism for attitude change, whose effect in certain contexts may outweigh the impact of structural biases. Future research could probe these competing dynamics more closely within specific sporting subcultures—for instance, professional leagues or traditionally male-dominated sports—to determine the conditions under which sports participation reinforces rather than challenges gender hierarchies.

Thirdly, the influence of sports participation on gender attitudes is comparatively weaker for men than for women. This phenomenon is related to the enduring social structures and cultural ideologies within the sporting realm. On one hand, long-standing institutional and symbolic inequalities within sports reduce both the external pressures and internal opportunities for men to re-examine their own gender roles. Empirical research indicates that the factual inequalities present in sports have a significant negative moderating association with the modernisation of men’s gender attitudes (Muñoz-Helú et al., 2025). For instance, gender preferences in the allocation of sports resources, gender-based streaming in early physical education, and media-perpetuated gender differences subtly reinforce men’s dominant position and sense of privilege within the sports domain (Lev, 2024; González-Calvo et al., 2025; Herrera et al., 2024). This structural advantage may diminish the potential of sports participation to challenge gender stereotypes, reducing men’s impetus and urgency—often experienced by women—to critically reflect on the broader gender power order through bodily practice, ultimately leading to a weakened practical effect. On the other hand, the deep entanglement of traditional hegemonic masculinity with sports culture may limit the diversification of men’s own gender attitudes. Sport has long been viewed as an institutionalized field for performing and consolidating dominant masculinity (Caldwell, 2023). Its rule systems, organisational practices, and cultural symbols collectively contribute to the reproduction of hegemonic masculinity, thereby potentially supporting men’s dominant status. Furthermore, the deep entanglement of traditional hegemonic masculinity with sports culture may also influence the diversification of men’s own gender attitudes (Adriaanse, 2013). Thus, the practical effectiveness of Sports participation in promoting the modernization of men’s gender attitudes may still require further enhancement.

## 6 Limitations and future directions

This study, by proposing a conceptual framework of “bodily experience—cognitive shift—social wellbeing,” provides a systematic theoretical lens for understanding the micro-level interaction between sports participation and individuals. However, the research acknowledges several limitations: First, due to limitations of the original questionnaire and database, this study did not directly operationalize or statistically examine mediating constructs such as “bodily experience” or “cognitive transformation.” This study primarily focuses on explicating and constructing the theoretical pathway. Research that identifies psychological mechanisms through experimental manipulation and precise measurement represents a cutting-edge paradigm shift from “correlational validation” to “mechanism testing.” Therefore, future research should adopt this paradigm to develop and validate measurement tools for constructs such as “bodily experience” and “cognitive reflection” in sport contexts, and employ longitudinal tracking or randomized intervention experiments to conduct more rigorous causal mechanism tests of the conceptual framework proposed in this study. Second, although this study improves the reliability of causal inference to a certain extent through the instrument variable of ‘attending live matches,’ providing valuable exploratory evidence for understanding the causal relationship between sports participation and gender attitudes, it is still necessary to acknowledge the limitation that the exclusion restriction assumption of the instrument variable is difficult to fully verify. That is, attending live matches may influence individuals’ gender beliefs through other channels (e.g., media narratives, public visibility of female athletes). Future research could further adopt more exogenous identification strategies, such as using policy shocks, or combine longitudinal data designs to more rigorously test the robustness of the findings of this study. Third, although this study systematically controlled for key observable confounders at the individual level, the cross-sectional nature of the data prevents us from fully ruling out the possibility of unobserved confounding factors. Future research could further validate the robustness of our findings by utilizing longitudinal data, natural experimental designs, or incorporating richer psychological and contextual measures.

## Data Availability

The datasets can be found at: Chinese General Social Survey (CGSS), http://cgss.ruc.edu.cn/.

## References

[ref1] Adriaanse J. A. (2013). “The role of men in advancing gender equality in sport governance,” in Gender and Sport: Changes and Challenges, 50–70.

[ref2] Anderson E. (2010). Inclusive Masculinity: The Changing Nature of Masculinities. New York: Routledge.

[ref3] Barragan R. (2015). Sport participation, gender schema, athletic identity, and internalized homophobia in lesbian women.

[ref4] Berg A. Duffy D. DuBois S. (2023). Elegant violence: the promise and peril of a new ‘feminine’ sport ethic. Sport Soc. 26, 335–349. doi: 10.1080/17430437.2022.2033223

[ref5] Bhardwaj S. (2022). Decision-making in the recruitment of women on corporate boards: does gender matter? Equal. Divers. Inclus. Int. J. 41, 813–830. doi: 10.1108/edi-08-2021-0188

[ref6] Blee K. M. Tickamyer A. R. (1995). Racial differences in men's attitudes about women's gender roles. J. Marriage Fam. 57, 21–30. doi: 10.2307/353813

[ref7] Brown D. (2005). An economy of gendered practices? Learning to teach physical education from the perspective of Pierre Bourdieu's embodied sociology. Sport Educ. Soc. 10, 3–23. doi: 10.1080/135733205298785

[ref9] Brown W. J. Mielke G. I. Kolbe-Alexander T. L. (2016). Gender equality in sport for improved public health. Lancet (London, England) 388, 1257–1258. doi: 10.1016/S0140-6736(16)30881-9 27475268

[ref10] Caldwell D. (2023). Enacting power, solidarity and masculinity in sport: a discourse analysis of the language-in-action of aboriginal boys. Int. J. Educ. Res. 121:102227. doi: 10.1016/j.ijer.2023.102227

[ref110] Cole C. L. (2000). Body studies in the sociology of sport: a review of the field. Handbook of Sports Studies, 439–460.

[ref11] Dai Y. (2023). The impact of rural-urban migration on rural youth's gender attitude and its heterogeneity——an empirical analysis based on CGSS 2012—2018 data. Agricultural University Journal of Social Sciences 40, 35–56. doi: 10.13240/j.cnki.caujsse.20230918.001

[ref12] Fan C. C. (2003). Rural-urban migration and gender division of labor in transitional China. Int. J. Urban Reg. Res. 27, 24–47. doi: 10.1111/1468-2427.00429

[ref13] Fink J. S. LaVoi N. M. Newhall K. E. (2018). “Challenging the gender binary? Male basketball practice players' views of female athletes and women's sports,” in Sex Integration in Sport and Physical Culture, (New York: Routledge), 206–221.

[ref14] Frankfort-Nachmias C. Leon-Guerrero A. Davis G. (2020). Social Statistics for a diverse Society. London: Sage publications.

[ref15] Gardella L. G. (2023). Origins of social group work: local stories from around the world. Soc. Work Groups 46, 285–288.

[ref16] González-Calvo G. Gerdin G. García-Monge A. (2025). Soccer: the only way to be a boy in Spain? Narratives from a Spanish primary school on the influence of playground soccer in shaping masculinities and gender relations. Sport Educ. Soc., 1–15. doi: 10.1080/13573322.2025.2459686

[ref17] Gorely T. Holroyd R. Kirk D. (2016). “Muscularity, the habitus and the social construction of gender: towards a gender-relevant physical education,” in Masculinity and Education, (New York: Routledge), 48–67.

[ref18] Guerrero M. A. Guerrero Puerta L. (2023). Advancing gender equality in schools through inclusive physical education and teaching training: a systematic review. Societies 13:64. doi: 10.3390/soc13030064

[ref20] Herrera A. Sánchez-Hernández M. D. Herrera M. C. Expósito F. (2024). Athlete portraits in news: influence of media representation and gender on social perception. Span. J. Psychol. 27:e26. doi: 10.1017/SJP.2024.21, 39463056

[ref21] Huggins A. Randell S. (2007). “The contribution of sports to gender equality and women’s empowerment,” in A Paper Presented at the International Conference on Gender Equity on Sports for Social Change, Kigali, vol. 3 (), 2009.

[ref22] Işıkgöz M. E. Şahbudak M. Deveci M. E. Öztunç M. (2025). Challenges and successes in promoting gender equality through physical education and sports: a systematic review. BMC Public Health 25:2117. doi: 10.1186/s12889-025-23373-0, 40481444 PMC12143033

[ref23] Jejeebhoy S. J. Acharya R. Pandey N. Santhya K. G. Zavier A. J. Singh S. K. . (2017). The effect of a gender transformative life skills education and sports-coaching programme on the attitudes and practices of adolescent boys and young men in Bihar.

[ref24] Lanhua Y. Yao C. (2021). Existential anxiety: multiple logics of modern female gender identity—based on CGSS 2015 data analysis 11, 90–97+159. doi: 10.14167/j.zjss.2021.11.010

[ref25] Lev A. (2024). From combat boots to running shoes: the role of military service in shaping masculine identity in Israeli long-distance running groups. Int. Rev. Sociol. Sport 59, 868–885. doi: 10.1177/10126902231226409

[ref26] Li W. (2021). Carding and reflection: the core rationale of Bourdieu sports practice research and its application in China. J. Jilin Inst. Phys. Educ. 37, 21–26.

[ref27] Lyras A. Hums M. A. (2009). Sport and social change: the case for gender equality. J. Phys. Educ. Recreat. Dance 80, 7–21.

[ref28] Mahony D. F. Hums M. A. Andrew D. P. Dittmore S. W. (2010). Organizational justice in sport. Sport Manage. Rev. 13, 91–105. doi: 10.1016/j.smr.2009.10.002

[ref29] Meier M. (2000). Gender Equity, Sport and Development. Biel: Swiss Academy for Development.

[ref30] Meng X. (2011). Sociology of body. J. Wuhan Inst. Phys. Educ. 45, 24–27. doi: 10.15930/j.cnki.wtxb.2011.09.007

[ref31] Meyer K. L. Roche K. M. (2017). Sports-for-development gender equality impacts from basketball programme: shifts in attitudes and stereotyping in Senegalese youth and coaches. J. Sport Dev. 5, 49–57.

[ref32] Moura E. S. (2021). “I can’t because I am a man”: masculinity, manhood, and gender equality in sport for development. Sociol. Sport J. 39, 231–239.

[ref33] Muñoz-Helú H. Reynoso-Sánchez L. F. Cruz-Morales K. N. Salazar-C C. M. Mataruna-Dos-Santos L. J. (2025). In the perception of the Olympic movement and gender equity in sport, are gender and sport practice determining factors? Front. Sports Act. Living 7:1564617. doi: 10.3389/fspor.2025.1564617, 40230377 PMC11994579

[ref35] Ogilvie M. F. McCormack M. (2021). Gender-collaborative training in elite university sport: challenging gender essentialism through integrated training in gender-segregated sports. Int. Rev. Sociol. Sport 56, 1172–1188. doi: 10.1177/1012690220980149

[ref36] Oxford S. (2019). ‘You look like a machito!’: A decolonial analysis of the social in/exclusion of female participants in a Colombian sport for development and peace organization. Sport Soc. 22, 1025–1042. doi: 10.1080/17430437.2019.1565389

[ref37] Pérez-Díaz L. Blázquez-Alonso M. Moreno-Manso J. M. Lucas-Milán M. G. Cantillo-Cordero P. García-Baamonde M. E. (2025). Cognitive strategies and social attitudes that perpetuate gender inequality in secondary education students. Soc. Sci. 14:388. doi: 10.3390/socsci14060388

[ref38] Pfister G. (2011). Gender equality and (elite) sport. Enlarged Partial Agreement on Sport, 51–66.

[ref39] Scheadler T. Wagstaff A. (2018). Exposure to women’s sports: changing attitudes toward female athletes. Sport J. 19, 1–17.

[ref40] Schultz J. (2018). Women's Sports: What Everyone Needs to Know®. Oxford: Oxford University Press.

[ref41] Shaw S. Frisby W. (2006). Can gender equity be more equitable?: promoting an alternative frame for sport management research, education, and practice. J. Sport Manage. 20, 483–509. doi: 10.1123/jsm.20.4.483

[ref42] Sport for Development and Peace International Working Group Secretariat (SDPIWGS) (2007). Literature Reviews on Sport for Development and Peace. Toronto: University of Toronto, Faculty of Physical Education and Health.

[ref43] Tännsjö T. Tamburrini C. (2000). Values in Sport: Elitism, Nationalism, Gender Equality and the Scientific Manufacture of Winners.

[ref44] The State Council Information Office of the People's Republic of China. (2015). Gender equality and women's development in China. [White paper]. Available online at: https://www.gov.cn/zhengce/2015-09/22/content_2936783.htm

[ref45] Thorpe H. (2009). Bourdieu, feminism and female physical culture: gender reflexivity and the habitus-field complex. Sociol. Sport J. 26, 491–516. doi: 10.1123/ssj.26.4.491

[ref47] United Nations (2003). Sport for Peace and Development: Towards Achieving the Millennium Development Goals. New York: United Nations Inter-Agency Task Force on Sport for Development and Peace.

[ref48] Vaquero-Cristóbal R. Mateo-Orcajada A. Dağlı Ekmekçi̇ Y. A. . (2024). Gender equity in sport from the perspective of European women athletes and sport managers, physical education teachers and sport coaches. Front. Psychol. 15:1419578. doi: 10.3389/fpsyg.2024.1419578 39184942 PMC11342088

[ref49] Vezzali L. Visintin E. P. Bisagno E. Bröker L. Cadamuro A. Crapolicchio E. . (2023). Using sport media exposure to promote gender equality: counter-stereotypical gender perceptions and the 2019 FIFA women’s world cup. Group Process. Intergroup Relat. 26, 265–283.

[ref50] Wang B. Zhu T. (2024). The use of internet and the change of youth gender concept in the context of digital transformation: an empirical analysis based on CGSS 2012-2021 data. China Youth Stud. 3, 51–59. doi: 10.19633/j.cnki.11-2579/d.2024.0034

[ref51] Xiong H. (2025). Intersection and mutual construction: the regeneration and paradigmatic expansion of the sociology of the body theory in sports sociology. Sports Res., 1–19. doi: 10.15877/j.cnki.nsic.20250520.001

[ref52] Xiong H. Shen G. Han X. (2025). From ‘misogyny’ to ‘gender equality’: the historical process and social implications of gender equality in the Olympic movement. J. Shanghai Univ. Sport 49, 10–26. doi: 10.16099/j.sus.2024.12.05.0006

[ref53] Xu Q. (2016). Trend, source and heterogeneity of the change of gender-role attitude in China: a case study of two indicators. Collect. Women's Stud. 3, 33–43.

[ref54] Xu Y. Hu Y. (2025). Overcoming segregation: a study on spatial reconstruction for women in strength training. J. Beijing Sport Univ. 48, 19–32. doi: 10.19582/j.cnki.11-3785/g8.2025.04.002

[ref55] Yan J. Wen Y. (2025). Sport for sustainable development in the cause of gender equality: formation, content, impact and implications of the United Nations consensus. J. Capital Univ. Phys. Educ. Sports 37, 47–56. doi: 10.14036/j.cnki.cn11-4513.2025.01.005

[ref56] Yang X. (2024). Battle of the sex & gender: the “female” boxers at the Olympic games Paris 2024 45, 15–20. doi: 10.13598/j.issn1004-4590.2024.05.006

[ref57] Yang J. Li H. Zhu G. (2014). Changing trends in gender outlook in China from 1990 to 2010. Collect. Women's Stud. 6, 28–36.

[ref58] Yin H. Lu D. Hu S. (2021). Enlightenment of Bourdieu’s practice theory to the research paradigm of sports social practice. Sports Sci. 42, 46–52. doi: 10.13598/j.issn1004-4590.2021.05.006

[ref59] Yuan X. Zhang H. Pan Y. Liu S. Chen P. (2022). Is gender inequality holding women back? Evidence from China comprehensive social survey CGSS2017. Hum. Resour. Dev. China 39, 112–130.

[ref60] Zhang Q. (2021). How to generate stadium space?—significance and limitation of Bourdieu’s field theory. J. Jilin Inst. Phys. Educ. 37, 21–26. doi: 10.13598/j.issn1004-4590.2021.03.003

